# Draft Genome Sequence of Azole-Resistant Aspergillus thermomutatus (Neosartorya pseudofischeri) Strain HMR-AF-39, Isolated from a Human Nasal Septum Abscess Aspirate

**DOI:** 10.1128/MRA.01444-18

**Published:** 2019-01-03

**Authors:** Maxime Parent-Michaud, Philippe J. Dufresne, Éric Fournier, Christine Martineau, Sandrine Moreira, Vincent Perkins, Louis de Repentigny, Simon F. Dufresne

**Affiliations:** a Department of Microbiology, Infectious Diseases and Immunology, Université de Montréal, Montréal, Québec, Canada; b Laboratoire de Santé Publique du Québec, Institut National de Santé Publique du Québec, Sainte-Anne-de-Bellevue, Québec, Canada; c Department of Food Sciences and Nutrition, Université Laval, Québec City, Québec, Canada; d Division of Infectious Diseases and Clinical Microbiology, Department of Medicine, Maisonneuve-Rosemont Hospital, Montréal, Québec, Canada; Vanderbilt University

## Abstract

Here, we present the draft genome sequence of Aspergillus thermomutatus (formerly known as Neosartorya pseudofischeri; strain HMR-AF-39/LSPQ-01276), a cryptic species of Aspergillus section Fumigati. This species is intrinsically resistant to antifungal azoles and is recognized as an agent of invasive aspergillosis among immunocompromised hosts.

## ANNOUNCEMENT

Invasive aspergillosis is a life-threatening fungal infection in immunocompromised patients (1–4). In 2005, the genome of Aspergillus fumigatus, the most frequent species causing aspergillosis, was sequenced and published (5, 6). Aspergillus section Fumigati includes multiple closely related species that are frequently misidentified as A. fumigatus (7–10). Within this group, Aspergillus thermomutatus is a known human pathogen (7, 11–19). It is considered intrinsically resistant to antifungal azoles, displaying higher MICs to members of this class than A. fumigatus (16, 20, 21).

At Maisonneuve-Rosemont Hospital, 1,186 clinical isolates of presumed A. fumigatus were collected from 2000 to 2013. A. thermomutatus strain HMR-AF-39/LSPQ-01276 was isolated in 2000 from a pus aspirate, collected from a hematopoietic stem cell transplant recipient with nasal septum abscess. Broth microdilution antifungal susceptibility testing was conducted using the reference CLSI methodology (22) and revealed elevated MICs to itraconazole (2 µg/ml), voriconazole (2 µg/ml), and posaconazole (1 µg/ml), compared with wild-type A. fumigatus sensu stricto (23). To gain insight on potential genetic differences which may explain A. thermomutatus azole resistance, we sequenced the whole genome of this clinically significant isolate.

Whole-genome sequencing was performed at the provincial public health laboratory. Conidia harvested from a freshly grown culture (in potato dextrose agar at 30°C for 5 days) were subjected to DNA extraction using the ZR fungal/bacterial DNA miniprep kit (Zymo Research Corp.). Libraries of the genome of HMR-AF-39/LSPQ-01276 were prepared with the Nextera XT kit (Illumina, Inc.). Paired-end sequencing was performed using a MiSeq sequencing kit V3 600 cycles in pair mode (2 × 300 bp) on a MiSeq sequencing system (Illumina, Inc.). A total of 19,175,446 raw reads were generated. A qualitative and quantitative control was made on the resulting reads using FastQC (version 0.11.5; default parameters). First, raw reads were cleaned using Trimmomatic (version 0.36; with parameters leading, 20; trailing, 20; slidingwindow, 4:20; minlen, 50; and Illuminaclip, NexteraPE-PE.fa:2:30:10) (24). Then, pairs of cleaned reads mapping onto the Aspergillus fumigatus mitochondrial genome were discarded. SPAdes (version 3.7.0; parameters, -k 77,99,127) (25) was used to assemble the nuclear reads into contigs on Calcul Québec servers (http://www.calculquebec.ca/). The resulting assemblies were filtered by an in-house Python script to remove weakly supported contigs with coverage less than 5× and length below 2 kb (https://github.com/EricFournier3/PythonGenomicTools). The absence of duplicated contigs was assessed by running dedup.sh (https://jgi.doe.gov/data-and-tools/bbtools/), and the absence of Illumina adapters was checked using the linux grep command. A second quality control on contigs was made using QUAST version 4.1, (26) which computes assembly metrics and detects possible misassemblies. Specifically, we compared the total length of contigs with that of A. fumigatus as the closest reference genome and ensured an *N*_50_ value greater than a preestablished cutoff of 70 kb. The final assembly contained a total of 647 contigs (*N*_50_, 93,306 kb) for a genome size of 30,945,974 bp and a G+C content of 48%.

The annotation was performed using pipeline funannotate version 1.4.2. First, funannotate removed repetitive contigs (with option <clean>) and masked repetitive elements (using option <mask> combined with the parameter –repeatmasker_species fungi). Then, it predicted genes using the Aspergillus fumigatus pretrained gene model from Augustus (27) (option <predict> with parameters –busco_seed_species aspergillus_fumigatus and –augustus_species aspergillus_fumigatus) and performed functional annotation on them using the InterProScan5 pipeline (option <iprscan> combined with parameter –m docker to run in a InterProScan docker version 5.31 to 70.0). It resulted in 9,702 predicted genes, from which 1,363 have functional annotation. The completeness of this annotation was assessed by running Busco (version 2.0; Dikarya data set) (28). Our annotation of A. thermomutatus was able to retrieve 1,220 proteins among Dikarya-conserved orthologs (93%), only 47 proteins less than A. fumigatus, the closest reference species.

This is the first report of the whole-genome sequence of Aspergillus thermomutatus. These data will prove useful in gaining a better understanding of virulence factors and genetic mechanisms of antifungal resistance of this species as well as other related clinically relevant species.

### Data availability.

This whole-genome shotgun project has been deposited at GenBank under the accession number NKHU00000000. The version described in this paper is the second version, NKHU02000000. Raw sequence data can be accessed using the SRA run number SRR8165488.
